# Symptomatic Anemia Due to Cameron Lesions: A Case Report

**DOI:** 10.7759/cureus.73131

**Published:** 2024-11-06

**Authors:** Karim Girgis, Samantha Gaetani, David T O'Gurek, Marna R Greenberg

**Affiliations:** 1 Department of Emergency and Hospital Medicine, Lehigh Valley Health Network, Allentown, USA; 2 Morsani College of Medicine, University of South Florida, Tampa, USA; 3 Department of Family Medicine, Lehigh Valley Health Network, Allentown, USA

**Keywords:** cameron lesions, gastrointestinal bleeding, hiatal hernia, iron deficiency anemia, shortness of breath (sob)

## Abstract

Cameron lesions are a unique and relatively rare cause of upper gastrointestinal bleeding that appears in the mucosa of the gastric body in the presence of a large hiatal hernia. These lesions can be a source of occult bleeding and subsequent chronic iron deficiency anemia (IDA) but may often be missed on initial endoscopy, requiring repeat studies to diagnose. Prompt treatment for Cameron lesions is necessary to avoid the high mortality rate associated with them. We describe the case of a 36-year-old male patient who presented to the emergency department (ED) with shortness of breath (SOB) and chronic IDA of an unknown cause in the presence of a large hiatal hernia. The endoscopy showed multiple linear erosions in the stomach consistent with Cameron lesions. The patient was discharged but returned to the ED two more times before ultimately having his hiatal hernia surgically repaired. Though rare, Cameron lesions may be considered in the differential for patients presenting with recurrent SOB or with chest or abdominal pain, combined with refractory anemia of an unknown cause.

## Introduction

Gastrointestinal bleeding is a common presentation in the emergency department (ED) and is associated with a wide variety of etiologies [1]. Cameron lesions, also known as Cameron ulcers, were first described in 1986 by Cameron and Higgins [2]. They are a unique and relatively rare cause of upper gastrointestinal bleeding that appears in the mucosa of the gastric body in the presence of a large hiatal hernia [2]. On endoscopic examination, the lesions appear as superficial, white, linear erosions on the crests of the inflamed mucosal folds at the site of diaphragmatic indentation [3]. These lesions can be a source of occult bleeding and subsequent chronic iron deficiency anemia (IDA). However, these lesions can often be missed on initial endoscopy and may require repeat studies to diagnose [4]. The differential for anemia is quite broad, encompassing malignancies, intestinal diseases, gynecological disorders, chronic diseases, and poor diets, among others. Therefore, Cameron lesions may not generally be at the forefront of most differential diagnoses for patients presenting with anemia of an unknown cause, which can ultimately lead to potentially fatal complications if left untreated. 

It is important to recognize these uncommon Cameron lesions and provide prompt treatment to avoid the severe complications associated with them, which may even be fatal [4]. Additionally, the need for more information regarding these Cameron lesions is necessary in determining the best form of treatment. While medical management is currently the first-line treatment, hernia repair has been shown to resolve anemia in 63% of patients with Cameron lesions in previous studies [5]. In this report, we describe the case of a young patient who presented to the ED with a broad differential diagnosis of shortness of breath (SOB) and chronic IDA of an unknown cause, in the presence of a large hiatal hernia on several occasions that was eventually diagnosed with and treated for Cameron lesions.

## Case presentation

A 36-year-old male patient presented to the ED for worsening SOB for one week. While the differential for SOB included many alternative diagnoses, including but not limited to pneumonia, venous thromboembolism, or cardiac etiology, his SOB had been occurring intermittently over the last several months, with dyspnea on exertion with associated fatigue and lightheadedness. He had developed melena (dark/black-colored stools) over the last week. He denied any fevers, chills, chest pain, abdominal pain, nausea, vomiting, cough, or phlegm. The patient also complained of right leg swelling for the past month, but no edema was noted on the physical exam. He denied any alcohol use, smoking history, or drug use. He had a past medical history of IDA, deep vein thrombosis (DVT), pulmonary embolism (PE), and gastroesophageal reflux disease secondary to a hiatal hernia diagnosed several years prior. He had a past surgical history of an inguinal hernia repair several years prior, which was noted to have provoked the DVT and PE. The patient’s DVT and PE were then treated with apixaban for six months, and apixaban was prescribed for an additional two months after he developed superficial vein thrombosis. The patient was no longer prescribed anticoagulants at the time of presentation. His current medications include pantoprazole and sucralfate.

Upon chart review, his past medical history revealed that the patient was initially diagnosed with IDA after presenting to the ED 11 months prior with SOB, melena, and hemoglobin of 4.1 g/dL (reference range 12.5-17 g/dL). At that time, no cause was identified for the patient’s IDA. An endoscopy was completed with no acute findings aside from a hiatal hernia, and a colonoscopy was completed showing diverticulosis but no bleeding. Several months later, the patient had a negative pill endoscopy. He presented to the ED again for fatigue and SOB about a month before this current presentation. He did have a drop in his hemoglobin at that time (10.3 g/dL) but was not admitted to the hospital. He was discharged home with a gastroenterology follow-up. Finally, several days before this current presentation, the patient visited the ED of an outside hospital system for abdominal pain, melena, and coffee ground emesis. A contrast-enhanced computed tomography of the abdomen and pelvis was completed at that time and showed a moderate hiatal hernia but no acute abdominopelvic pathology.

On examination during this visit, the patient’s initial vital signs were a blood pressure of 119/75 mmHg, a heart rate of 85 beats per minute, a temperature of 98.7 °F (37.1 °C), a respiratory rate of 18 breaths per minute, and an oxygen saturation of 100% on room air. His physical examination showed a normal heart rate with normal heart sounds. His pulses were equal in all extremities. His lungs were clear to auscultation. He had no abdominal tenderness, distension, or palpable abdominal masses. He had normal bowel sounds. There was no lower extremity swelling. His rectal examination showed no active rectal bleeding or hemorrhoids. He had a good rectal tone. The stool was hemoccult positive.

**Figure 1:**
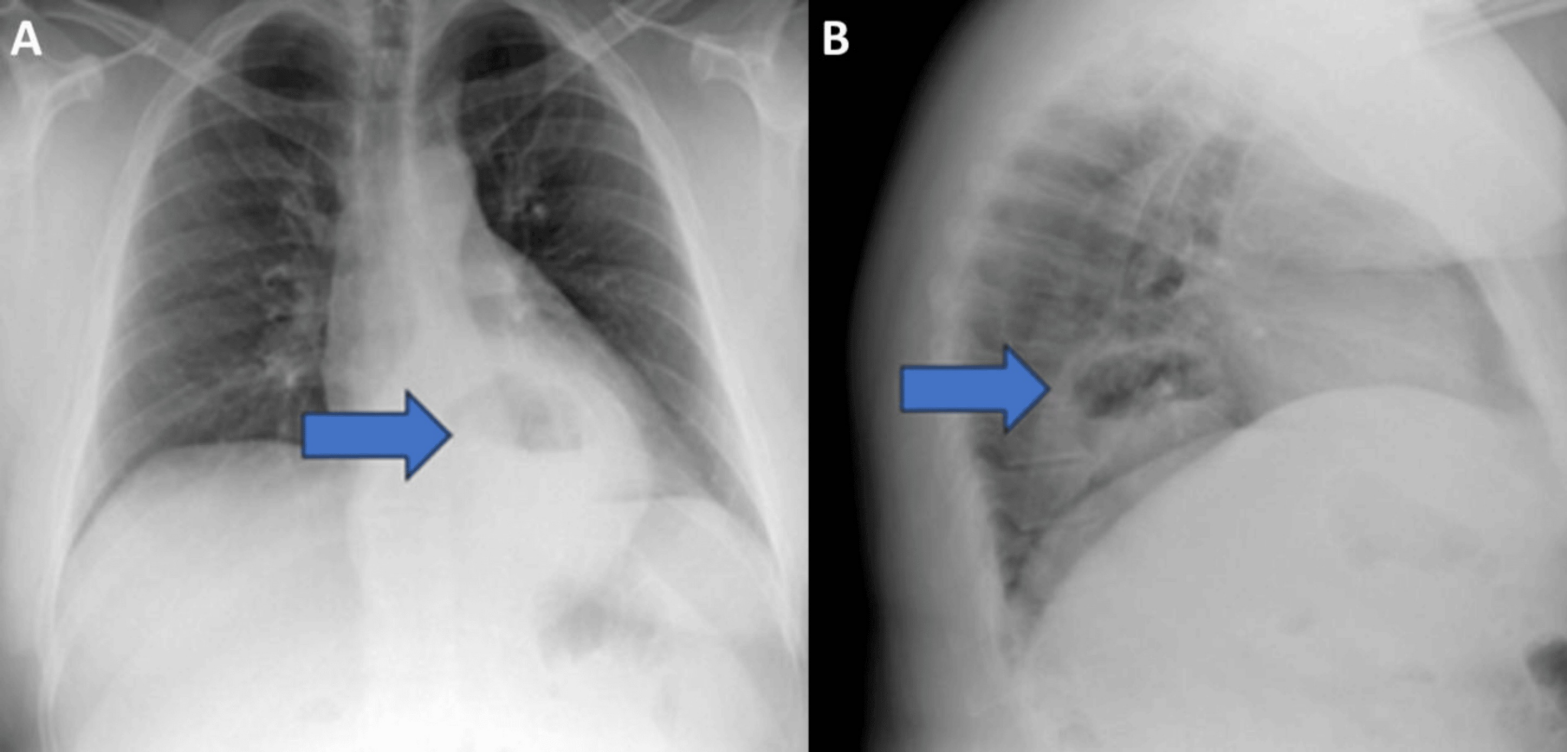
Chest X-ray, posterior-anterior view (A) and lateral view (B) The radiologist’s impression was a large hiatal hernia, which may be slightly larger than prior. No consolidation was seen. The arrows point to the large hiatal hernia.

**Figure 2:**
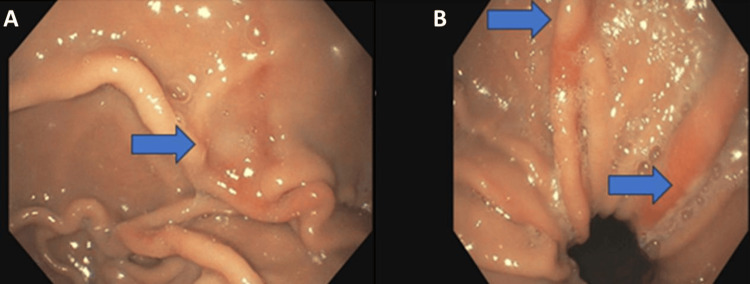
Cameron lesions in the stomach These two images (A and B) were obtained during endoscopy. The blue arrows in both images point to the Cameron lesions.

Abnormal ED laboratory results included a hemoglobin of 7.4 g/dL (reference range 12.5-17 g/dL), hematocrit 24.8% (reference range 37%-48%), platelets 385,000/mm^3^ (reference range 140-350,000/mm^3^), mean corpuscular volume 62 fL (reference range 80-100 fL), mean corpuscular hemoglobin 18.4 pg (reference range 27-36 pg), mean corpuscular hemoglobin concentration 29.9 g/dL (reference range 32-37 g/dL), red cell distribution width 22.8% (reference range 11.8%-14.5%), iron 15 ug/dL (reference range 50-212 ug/dL), and ferritin 2.9 ng/mL (reference range 23.9-336.2 ng/mL). The electrocardiogram showed normal sinus rhythm with no abnormalities. A chest X-ray was completed and showed a large hiatal hernia (Figure 1). The patient was admitted to the hospital for further work-up and management. He did receive one unit of packed red blood cells and intravenous iron infusions on this admission. A gastroenterologist performed an endoscopy that showed multiple linear erosions in the stomach consistent with Cameron lesions secondary to the patient’s large hiatal hernia (Figure 2). The patient was then seen by a general surgeon with plans for hernia repair on an outpatient basis. He was discharged from the hospital. Prior to his surgical repair, he returned to the ED again two more times for melena with SOB. He received further blood transfusions on these admissions. The patient ultimately had his hiatal hernia repaired robotically and postoperatively is doing well and following with general surgery. His anemia resolved after surgery.

## Discussion

The prevalence rates of hiatal hernia range from 0.8 to 2.9 in all patients undergoing upper gastrointestinal endoscopy, and Cameron lesions are seen in 5% of patients with known hiatal hernia discovered in upper endoscopic studies [6,7]. In this particular case, it was fairly unusual for such a young patient to present with a history of chronic anemia to such a severe degree with no known underlying cause despite repeated work-ups. It has been documented in early studies evaluating this unique diagnosis that these lesions were seen in people who had a chest X-ray showing one-third or more of the stomach above the diaphragm, and they reported that of the total cases, 50% were found to be anemic [2,8]. Since Cameron lesions can be missed on initial endoscopy, repeat procedures are often necessary for refractory undiagnosed cases and are very helpful in determining the diagnosis. Our patient similarly did not have the diagnosis made on the initial diagnostic evaluations. In previous studies, it was found that about 69% of patients had undergone one or more previous upper endoscopy studies before being diagnosed with a Cameron lesion [4].

Additionally, seeing as this type of gastric ulcer is quite uncommon, many clinicians might not immediately consider it as a part of the differential upon presentation. It is necessary to rule out life-threatening conditions such as pneumonia, PE, and aortic dissection, among others. After excluding these lethal diagnoses, the focus can be placed on rarer diseases with similar presentations.

Oftentimes, acid suppressants are the drugs of choice for the medical management of Cameron ulcers, as well as iron supplementation for accompanying anemia [9]. Many patients with Cameron lesions may already have a history of reflux disease secondary to long-standing hiatal hernias and already be taking proton pump inhibitors before the official diagnosis is made, as was seen in our case. In such cases, intensification of the treatment is recommended [9]. However, about 20% of patients may have persistent severe anemia and rebleeding which prompts surgical treatment with retraction of the hernia, closure of the weakness in the diaphragm, and fundoplication [10]. Considering the presenting symptoms of the patient described in our case, surgical treatment was seen as the most beneficial solution. After the surgical treatment, our patient responded well and had a resolution of the refractory anemia.

## Conclusions

It is necessary to rule out life-threatening conditions for patients presenting with SOB and chest or abdominal pain. After excluding these lethal diseases, broadening the differential to include rarer conditions can be helpful. Though rare, Cameron lesions may be the culprit in patients presenting with SOB or with chest or abdominal pain, especially those with anemia of an unknown cause. This can be especially important in patients who may be younger than expected or presenting with more severe anemia than expected for their current findings such as in the case we discussed. 

This case demonstrates the importance of recognizing these rare Cameron lesions in patients with refractory anemia to appropriately treat and manage bleeding before complications arise. Clinicians may be alerted to this potential diagnosis by the appearance of the chest X-ray, in particular, if part of the stomach is above the diaphragm. Eventual diagnosis of Cameron ulcers is especially important to appropriately treat the disease.
